# Vascular Changes and Hypoxia in Periodontal Disease as a Link to Systemic Complications

**DOI:** 10.3390/pathogens10101280

**Published:** 2021-10-05

**Authors:** Dilek Celik, Alpdogan Kantarci

**Affiliations:** 1 Immunology Division, Health Sciences Institute, Trakya University, Edirne 22100, Turkey; celiknd@outlook.com; 2 Forsyth Institute, Cambridge, MA 02142, USA; 3 School of Dental Medicine, Harvard University, Boston, MA 02142, USA

**Keywords:** periodontal disease, inflammation, hypoxia, vascular changes

## Abstract

The hypoxic microenvironment caused by oral pathogens is the most important cause of the disruption of dynamic hemostasis between the oral microbiome and the immune system. Periodontal infection exacerbates the inflammatory response with increased hypoxia and causes vascular changes. The chronicity of inflammation becomes systemic as a link between oral and systemic diseases. The vascular network plays a central role in controlling infection and regulating the immune response. In this review, we focus on the local and systemic vascular network change mechanisms of periodontal inflammation and the pathological processes of inflammatory diseases. Understanding how the vascular network influences the pathology of periodontal diseases and the systemic complication associated with this pathology is essential for the discovery of both local and systemic proactive control mechanisms.

## 1. Introduction: Overview of the Vascular Impact of Periodontal and Oral Inflammation

Physical barriers are the first lines of defense against pathogenic microorganisms. The skin covering the outer surface of the body and the mucosal surfaces covering the internal spaces prevent external agents, including the microorganisms, from penetrating deeper tissue compartments and causing disease in the host. This notion primarily applies to bacteria, viruses, and other “foreign” organisms as opposed to physical and chemical agents. The human body hosts ten times more bacteria than its own cells at any given time and therefore has its own bacterial species living in complex microbiome communities. The mouth is among the most microorganism-populated environments in the human body, where the mucosal surfaces come into contact with the highest number and variety of microbial species [1]. As shown by the Human Microbiome Project [2], mutual and pathogenic bacteria groups in the oral cavity present a complex environment where salivary proteins, glycoproteins, epithelial cell debris, and food debris contribute. Thus, there is a need for a highly specialized immune surveillance network in such a complex environment to modulate and stabilize interactions between the host and pathogenic or commensal bacteria [3]. The oral mucosal immune network is specialized in tissue-specific immunological tolerance, tissue homeostasis, interactions with microbiome species, and developing defense.

Immune system balance is critical for a sustained host response. While an inadequate immune response would facilitate infection, an excessive inflammatory response due to an overreacting immune system would cause tissue damage to the self [4]. Inflammation appears to be tissue-specific; however, it can lead to pathologies in distant tissues or organs when the process becomes chronic. It is, thus, plausible that there is a link between inflammatory diseases, considering that mediators, cells, and mechanisms play a similar role in local and systemic processes. The earliest evidence for such a link was demonstrated between periodontal diseases and diabetes as a bi-directional association [5]. As a result of impaired insulin metabolism in diabetes mellitus, the hyperglycemic environment around the vessels may cause uncontrolled inflammation. There is also evidence that inflammation associated with periodontal disease adversely affects glycemic control [6,7,8]. In patients with diabetes and periodontitis, systemic inflammation caused by an oral infection may thus affect vascular endothelial cells with free oxygen radicals and inflammatory mediators. As a result, endothelial cells exposed to oxidative stress may contribute to diabetes pathologies with increased insulin resistance, suggesting that a link between periodontal diseases and systemic pathologies may be through vascular dysfunction and mediated by endothelial cells. Indeed, later studies demonstrated that cardiovascular diseases associated with atherosclerotic changes might be exacerbated by periodontal infection and inflammation, emphasizing the vascular link as a potential pathogenetic mechanism where oral pathogens were detected in atherosclerosis plaques [9]. 

Bacteremia, which impairs vascular hemostasis, can cause a series of reactions that cause the release of prothrombotic mediators from endothelial cells and shift vascular tone in the vasoconstrictive direction [10]. As a result, lipid peroxidation increases and helps develop atherosclerotic plaque [11,12,13]. The oral pathogens can modify low-density lipoproteins [14], activating prothrombotic factors and promoting foam cell formation in the vessel. Decrease in nitric oxide release, oxidative stress, and increase in angiotensin II causes loss of function in the vessel. An inflammatory response occurs in the vessel, the vessel’s destruction, and plaque formation on its surface. In particular, the decrease in nitric oxide bioavailability triggers inflammation from endothelial cells. 

The relationship between atherosclerosis and periodontitis involves the release of periodontal disease-related pro-inflammatory cytokines and acute-phase proteins in circulation, changing vascular endothelial cell function. Studies have shown that patients with atherosclerosis and periodontitis have similar cytokine profiles [1,15,16,17] with high pro-inflammatory cytokines such as IL-6 [18], TNF-α [19], and acute-phase proteins (e.g., CRP) [18,20] in serum. These inflammatory mediators can trigger endothelial cell activation, leading to vascular inflammation, mononuclear cell inflammation, adhesion molecule expression, platelet aggregation, foam cell formation, and atherosclerotic plaque formation. Strong evidence for this link comes from studies that showed a decrease in early atherosclerotic plaque formation and systemic inflammation after periodontal treatment [21,22]. 

Rheumatoid arthritis (RA) and chronic periodontitis also have similar pathological processes. In both diseases, chronic inflammatory process, soft and hard tissue destruction, the release of pro-inflammatory cytokines and immune cell inflammation during active periods of disease, osteoclast differentiation, and maturation by RANK-L pathway activation (nuclear factor kappa B-ligand) are observed [23,24]. In addition, both diseases cause an increase in serum CRP and systemic inflammation [19,25] responsible for tissue destruction. In the context of RA, a “two-hit” mechanism was suggested to explain its association with periodontal disease [1]. According to this theory, the first hit is that the oral pathogen initiates the pathogenesis of RA by impairing tolerance. The second hit involves systemic inflammation that increases circulating biomarkers associated with oral bacteria. This inflammatory induction is thought to increase the activation of cells, causing tissue destruction and osteoclast differentiation and activity in both the synovium and oral tissue. Oral pathogenic bacteria can enter the circulation and reach the synovial fluid as shown by detecting *Porphyromonas gingivalis* [25], *Treponema denticola, Prevotella intermedia, Prevotella nigrescens, Tannerella forsythia*, and *Fusobacterium nucleatum* DNAs in synovial fluid of RA patients [26]. In particular, the contribution of *P. gingivalis* to the pathogenesis of RA has been demonstrated [27,28,29]. *P. gingivalis* can form neoepitopes through antigen mimicry, one of the escape mechanisms of bacteria from the immune system [30]. These epitopes cause citrullination of peptides at the protein folding stage using the peptidyl arginine deiminase (PAD) enzymes [31]. *P. gingivalis* citrullination presents its proteins and host proteins as new epitopes to the RA-specific genetic risk factor HLA DRB1*01 and DRB1*04 alleles. After antigen presentation, the immune system will initiate a reactive T cell response specific to autoantigens. These cells would then migrate to the synovial fluid and initiate the pathogenesis with the formation of autoantibodies. As a result, local inflammation may progress in the periodontium and joint capsules of RA patients via Fc and C5a receptors [32].

Early studies on the link between IBD (inflammatory bowel disease) and periodontal diseases suggested that mucosal inflammation derives from common pathogenesis in both processes [33,34]. IBD is characterized by invasion of commensal bacteria into the subepithelial space or lamina propria and has two major forms, ulcerative colitis (UC) and Crohn’s disease (CH). Bacterial invasion triggers a strong and unregulated inflammatory response that leads to increased epithelial permeability, intense neutrophil infiltration, and mucosal damage with impaired hemostasis. There are two hypotheses for the loss of immune tolerance to commensal bacteria. The first of these is a genetic predisposition that leads to an irregular and unbalanced mucosal immune response. The second is the changes in the intestinal microbiota composition that will provoke the normal inflammatory response. It is likely that both hypotheses contributes to the pathogenesis. Although the linkage mechanisms between periodontal disease and IBD are not yet fully understood, bacterial etiology, formation of dysbiosis, and the mechanisms of the similar immune response against dysbiosis suggest a relationship between the two diseases. Recent studies have provided substantial evidence for this association [35]. Comparing CD and UC patient microbiome and microbiome samples from healthy individuals, it was shown that oral pathogens *Firmicutes* and *Fusobacteria* were dominant in patient samples, but the normal flora bacteria *Bacteroidetes* and *Actinobacteria* were found in healthy individuals [36]. The entry of bacteremia from the oral cavity, albeit at a low level and chronic, into the system and especially the enteral spread of gastric acid-resistant oral pathogens cause colonization of oral bacteria located in the gut [37]. Moreover, in periodontitis, it is a possible mechanism that pathogens migrate to the intestine via the lymphatic route with a predominant Th17 response and then exacerbate inflammation with similar cytokine network and immune response profiles after causing microbiome changes [37,38,39]. The oral pathogen *P. gingivalis* given orally to subjects has been found to alter the composition of the gut microbiota, impairing serum metabolites and intestinal barrier function [40]. *F. nucleatum* is the most common strain in the altered microbiome of patients with IBD, and patients with *F. nucleatum*+ CD are significantly predisposed to settle in the gut [36]. Concomitantly, *F. nucleatum* has been shown to be an important risk factor for colorectal cancer and associated IBD [41]. In 10–90% of patients with colorectal cancer, *F. nucleatum* is found in tissues where *F. nucleatum* induces the growth of colorectal cancer cells by activating Wnt/β-catenin signaling [42].

Another level of evidence for the inflammatory link between periodontal disease and systemic diseases comes from recent studies on Alzheimer’s disease. Alzheimer’s disease is characterized by the accumulation of amyloid-beta proteins, an increase in tau fibrils. Although the pathogenesis of Alzheimer’s is thought to be dependent on age and genetic factors, it has been suggested that periodontal diseases induce or exacerbate Alzheimer’s disease [43,44]. Bacteremia and an increase in circular biomarkers, particularly CRP and IL-6 [45,46], can cause changes in the blood-brain barrier and activation of resident and inactive microglial cells in the brain [47]. It has been shown that bacteria or their toxins can enter the brain tissue and settle in amyloid plaques [48]. Immune activation of neurovascular network endothelial cells may aggravate inflammation by attracting systemic neutrophils and monocytes to the area. Astrocytes and microglial cells near the vessels may contribute to this inflammatory cascade, increasing the production of amyloid β40-42 proteins [47]. Thus, the production of amyloid protein is a form of immune defense tool, but tissue composition deterioration and damage may be inevitable due to uncontrollable inflammation. Therefore, it can be argued that chronic systemic inflammation will play an important role in the pathogenesis of the disease over the years.

Collectively, local inflammation generated by periodontal infection becomes chronic and systemic as a link between oral and systemic diseases [49]. Meanwhile, periodontitis, as an inflammatory disease, presents with an impaired immune regulation [49,50,51,52], where inflammation-based therapeutic approaches provide a resolution for both periodontal diseases and systemic inflammatory diseases in the same host, further demonstrating the central role of inflammation as a link between local and systemic diseases [53,54]. The vascular network plays a central role in this process, directing the immune response and regulating the activation and resolution of inflammation. A few studies have shown how vascular cells affect paracrine and autocrine and how this interaction affects systemic complications. This review focuses on local and systemic vascular network change mechanisms of periodontal inflammation and pathological processes of inflammatory diseases. 

## 2. Host Immune Response to Oral Bacteria

The first defense components against oral microorganisms are the epithelial barriers, saliva, complement system in the gingival crevicular fluid, and neutrophil cells below the epithelium. Saliva contains several antimicrobial agents (immunoglobulins such as IgA, IgM, IgG, and antimicrobial peptides such as histatins, lysozyme, lactoferrin, peroxidases) [55]. The periodontal disease begins with the invasion of oral bacteria into the gingival tissue. The first response of the rich gingival vascular network and tissue to bacterial invasion is vasodilation followed by increased permeability of blood capillaries, increased blood flow, increased vascularity, and infiltration of inflammatory cells. 

The majority of immune cells in the healthy gingival sulcus are neutrophils, representing approximately 95% of the total leukocytes and increases in inflammation [56]. Moutsopoulos and coworkers have performed a cohort study to evaluate the distribution of immune cells and their subsets in the inflamed tissues of patients with severe bone loss and previously untreated periodontitis [57]. In healthy samples, CD3^+^ T cells were the densest among lymphocyte cell groups; B cells (CD19, CD20) were minimal. CD1a^high^ (EPCAM^+^) cells and CD4^+^ helper T cells dominated, followed by CD8^+^ helper T cells and γδT cells. There was a significant increase in B cells in the samples with periodontitis compared to the healthy group. In addition, Th17 cells were also detected in the samples of patients with chronic periodontitis [58,59].

The most recent classification of periodontal and peri-implant diseases introduced and established stages as a basis for recognizing the progressive pathogenetic mechanisms underlying the periodontal diseases. While the information associating each stage with a specific immunological and vascular profile is scarce and heavily based on pathological specimens obtained from biopsy or surgical materials, evidence supports the new classification at the biological level. However, the progression of periodontal disease is not always linear and continuous. There are also several distinct forms of periodontal diseases, such as molar/incisor pattern (previously referred to as local aggressive periodontitis), which has stronger links to genetic and ethnic factors than the other forms of periodontitis. In addition, it is clinically impossible to test whether all gingivitis cases will predispose to periodontitis. Finally, evidence mounted for the immunological basis of periodontitis was heavily derived from Stage 3 and 4 periodontitis, which requires further research in the initial stages of periodontitis in the context of immunological pathways of inflammation. Nevertheless, the most recent classification set a premise for understanding chronic inflammation as a progressive process underlying periodontal diseases, which will be the basis of this review.

As in other forms of chronic inflammation, innate immunity is activated in the initial phase of lesion formation against pathogens. In this stage, where there is a non-specific response to the pathogen, cells of local epithelial origin initiate a response with local neural cells, immune system cells, complement, fibrinolytic, and kinin systems, both independently and in association with each other. Recognition of the pathogen is the most critical step of defense. Pathogen-specific patterns are detected by specialized PRRs in phagocytic, dendritic, and epithelial cells located at the entry sites of bacteria and lead to activation of the inflammatory cascade. PRRs are evolutionarily conserved extracellular or intracellular non-self-recognition tools that bind to the common structures of pathogens (pathogen-associated molecular patterns (PAMPs). Oral bacteria that migrate from the surface biofilm to the subepithelial tissue during oral infection are detected by the PRRs of tissue-resident neutrophils, macrophages, and epithelial cells, activating the innate immune response. The most well-characterized PRRs are the TLR-2 (Toll-like receptor-2) and TLR-4, which play a key role in bacterial recognition [60,61]. PRRs also include lectin and scavenger receptors, nucleotide-binding oligomerization domain (NOD) proteins, CD14 protein, non-cell-mediated and circulating complement receptor-3, mannose-binding lectins, and C-reactive proteins.

Meanwhile, neuropeptides are released that will trigger the vascular network with neuron stimulation and initiate lymphocyte migration, as well as the complement system, a humoral response component of the innate immune system, is activated. The complement system is involved in the phagocytosis of pathogens, damage to the bacterial membrane, and triggering the innate immune response. They costimulate the innate immune response with PRRs. The complement components in the gingival crevicular fluid in the oral cavity activate the alternative pathway of the complement system against oral pathogens. The C3a and C5a anaphylatoxin components of the alternative pathway induce chemotaxis and release vasoactive amines, free oxygen intermediates, and pro-inflammatory cytokines TNF-α, IL-1, IL-6 from phagocytes [49]. In addition, the C5b-C9 membrane attack complex, which is the end product of the pathway, is involved in the destruction of the bacterial membrane. 

Neutrophils, macrophages, and T cells produce a controlled and moderate immune response against plaque biofilm. After recognizing pathogens, the release of chemoattractants and a pro-inflammatory cytokine network are initiated in the region. Endothelium, activated by TNF-α, IL-1, IL-6 pro-inflammatory cytokines released from mononuclear cells, increases lymphocyte infiltration by directing the response to the inflammation site. At this stage, phagocytic cells—tissue-resident macrophages and neutrophils—are active, and neutrophils infiltrate the area in the first hours of inflammation. Degranulation of neutrophil toxins has a rapid but brief effect in clearing pathogens. Degranulation also causes the destruction of tissue cells and extracellular matrix. Both the destruction of the complement system by the pathogens on the cell surface and the phagocytosis of the pathogens it opsonizes by phagocytic contribute to the destruction of pathogens. C3a and C5a activate the mast cell, inducing the release of the pro-inflammatory cytokine and vasoactive amines (histamine and leukotriene-4). These vasoactive substances initiate phagocyte chemotaxis by expressing adhesion molecules and chemokines from endothelial cells and increasing vascular permeability and leukocytes migration [62]. The neutrophil response acts as a sensitive bridge between innate immunity and adaptive immunity. From the first hours of the innate response to Day 3, neutrophil cells can regulate both inflammation and resolution [63]. If neutrophils eliminate the pathogens, the innate reaction will be suppressed, and phagocytosis of cell debris will begin. Neutrophil granule proteins have been shown to act as pro-inflammatory cytokine suppressor and anti-inflammatory signal modifier in immunomodulation [64]. In addition, phagocytosis of apoptotic neutrophil causes the production of anti-inflammatory cytokines such as IL-4, IL-10, IL-12, and TGFβ at the resolution stage of inflammation and shifting of M1 macrophage (IFNγ-mediated innate response) to M2 macrophage (anti-inflammatory effect) [55] also induce [56,57]. Meanwhile, the acute inflammatory response is inhibited, and the biosynthesis of lipid mediators-lipoxins and resolvins (LXA4, resolvin E1, resolvin D1, maresin-1, and protectin D1) is increased in the repair of tissue damage. However, in cases where the infection cannot be eliminated, and the lesion enlarges, the cross-talk of tissue neutrophils, macrophages, and resident Langerhans cells (of similar phenotype in the skin) initiates the secondary phase initiates the adaptive response. Langerhans cells and macrophages from the innate immunity-associated antigen-presenting cells migrate to the nearest lymph node to present the bacteria-specific antigen [65]. In the oral mucosa, Langerhans cells play a role in the selection of specific naive T cells. Naive T cells come to the area of inflammation, become activated, and proliferate. CD4+T cells and antigen-presenting cells initiate the adaptive response. Innate immunity-associated antigen-presenting cells migrate to the nearest lymph node, and after antigen presentation, specific CD4+T cell selection determines whether the response class should shift towards Th1, Th2, Th17, or Treg. The cytokine signaling network determines the direction of the response. Each response class plays a different protective role and contributes to immunopathology. Th1 cells interferon-γ, interleukin-2; Th2 cells are inter-leukin-4, interleukin-5, interleukin-6, interleukin-10, interleukin-13; Th17 cells interleukin-6, interleukin-21, and interleukin-23; Treg cells are characterized by the release of trans-forming growing factor-β, interleukin-2 cytokines [59]. The regulation of the response is determined by a complex cytokine network. Th1 cytokine class IFNγ increase macrophage stimulation and antibody-dependent cell-mediated cytotoxicity, producing a phagocytosis-dominated, cell-mediated immune response. IL-12 is an active stimulator of IFNγ production and therefore plays a role in regulating the response class. The basic cytokine of the Th2 response, IL-4, suppresses the release of IFNγ, causing the response to change from the Th1 direction to the Th2 direction. In this respect, IL-4 appears as a cytokine that both activates the Th2 response and regulates the response class. Likewise, IL-10 reduces the expression of IL-12 and B7 costimulatory receptors of antigen-presenting cells and inhibits cell proliferation. The Th17 response class stimulates the humoral response. T-regulatory cells have a protective role against periodontal tissue damage by expressing the glucocorticoid-inducible tumor necrosis factor receptor (GITR), the inhibitory molecule cytotoxic T-lymphocyte-associated molecule 4 (CTLA-4) and cell surface TGF-β1.

The adaptive response is controlled by three mechanisms: (i) APC modulation: T cell activation-costimulatory T cell anergic is made by suppressing CTLA-4, the APC inhibitor ligand of CD28 receptor; (ii) apoptotic death of lymphocytes in clearing the infection: Apoptotic death of lymphocytes is an extremely important mechanism of immune regulation. Elimination of pathogen antigens by clearing the infection deprives immune cells of vital signals such as growth factors. Active cells undergo apoptosis with the programmed cell death mechanism in which caspase-9 and effector caspases are stimulated by the release of intracellular cytochrome c and apoptosis activating factor-1. TNF-mediated activation-induced cell death mechanism is another inhibition mechanism of active cells. Fas receptor (CD95)-FasL interaction on the T cell surface stimulates the caspase cascade through caspase 8 and causes apoptotic death of the cell; (iii) proliferation and activation of the T cell occur with stimuli between the antigen-presenting cell and the T cell. T cell costimulatory protein CD28-B7 interaction is essential for T cell activation. The APC inhibitor ligand CTLA-4 (antagonist B-7) suppresses the CD28 receptor and makes the T cell anergic.

If the pathogens have been eliminated, cellular debris is formed after the adaptive immune response is cleared by phagocytosis, and immune cells will increase the anti-inflammatory response by changing the cytokine network response class. As a result, both tissue homeostasis and vascular homeostasis are restored. However, in cases where periodontal inflammation continues, microbial plaque growth is prolonged, and the lesion expands.

The inflammatory lesion covers no more than 5–10 percent of the connective tissues at this initial stage and is still not clinically evident [66]. The response is reversible when the biofilm is removed, the immune response is modulated, and homeostasis is restored. However, the persistence of the biofilm increases the immunological response and causes it to become chronic.

**Figure 1:**
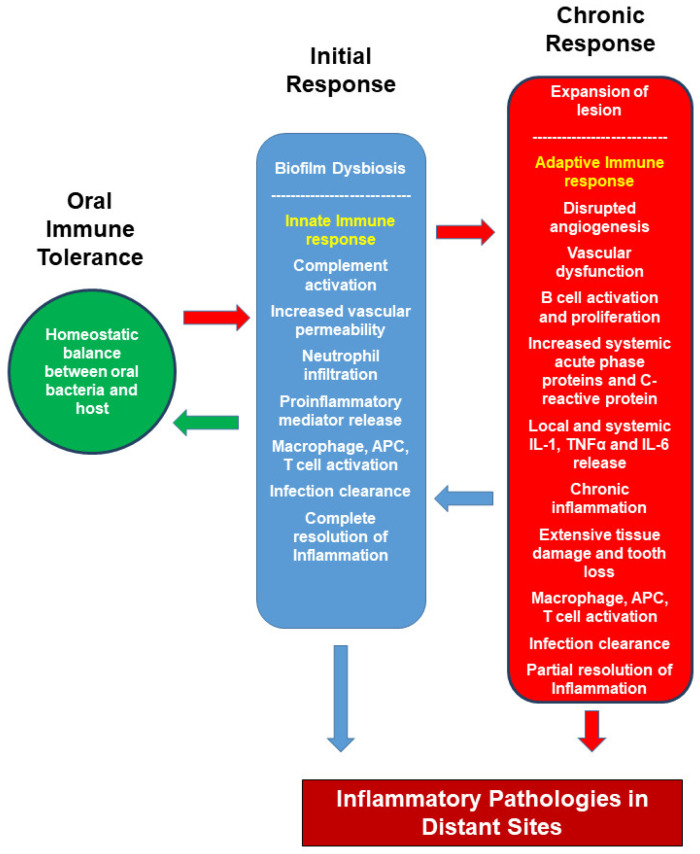
The immune response of periodontal diseases. There is a homeostatic balance between gingival bacteria and the immune system in the oral cavity, modulated by the immune system. The invasion of bacteria into the gingival tissue disrupts the delicate relationship, and an initial response develops in the gingival tissue. The bacterial invasion activates the innate immune system. In the early stage, the immune response is reversible and at a level where the damage caused by inflammation can be repaired. Inflammation does not persist if the lesion is removed. This stage is compatible with the pathogenesis of early gingivitis. Early gingivitis is the stage in which the invasion of dental plaque bacteria stimulates the innate immune response into the tissue, and acute vasculitis occurs when blood vessels dilate, and plasma components pass into the connective tissue compartment. With the activation of the endothelium, neutrophil infiltration begins in the inflammatory region. The host responds to these signals with saliva and the production of antimicrobial peptides to eliminate microorganisms. If the plaque cannot be removed, it can cause to establish gingivitis, inflammation exacerbation, and lesion establish tissue edema. In the following period, increasing the number and diversity of plaque bacteria causes an increased number of neutrophils, monocytes, and macrophages, T and B cells to migrate to the area of inflammation. Acute-phase proteins such as CRP, serum alpha amyloid A, and fibrinogen also increase blood levels. At this stage, teeth cleaning and plaque removal at this stage, tooth cleaning and plaque removal can revert gingivitis and restore periodontium to a healthy stage. However, if the infection persists, inflammation increases tissue destruction, lesion formation and enlargement are observed. The accompanying vascular dysfunction may cause the inflammation to spread and even become systemic. At this stage, the disease has transformed from gingivitis to periodontitis. Continued bacterial invasion introduces bacteria and their products into the systemic circulation, making both infection and inflammation chronic and aggravating. Periodontitis is characterized by chronic inflammation, extensive tissue damage, and tooth loss. Pro-inflammatory cytokines, acute-phase proteins, and CRP levels increase in serum, and inflammation spreads to distant sites by circulation.

The stabilization of the early lesion, IL-6, IL-1, and TNF-α pro-inflammatory cytokines collectively lead to a severe response. With the release of these cytokines, the production of acute-phase proteins and activation of the complement system and platelets occur—the levels of serum proteins such as CRP, serum amyloid A increase in the circulating blood [66]. Myeloid-derived monocyte/macrophage cells from the blood migrate to the site of inflammation, playing an essential role in regulating both effector cells and immune response. The release of macrophagic TNF-α and IL-1 induces chemokines and adhesion molecules (ICAM-1, VCAM-1, LFA-1, E-selectin, P-selectin) from the vascular endothelium. As a result, additional leukocyte groups migrate to the region. In an in vivo model of ligature-induced periodontitis, macrophages were shown polarized to M1 from M2 by *P. gingivalis* lipopolysaccharide (LPS) stimulation [67]. The indicator of progressive and chronic inflammation is the presence of plasma cells in the lesion. The chronic inflammation process of periodontitis from biofilm formation to tooth loss is shown in Figure 1. 

## 3. Local Inflammation and Vascular Changes

The peptidoglycans of bacteria in the oral biofilms, lipoteichoic acid, LPS penetrate the deeper tissues due to several destructive protease enzymes of microbial or host origin that destroy the epithelial surface and periodontal ligament. These enzymes contribute to the deepening of the dental pocket and disrupt the endothelium of the subepithelial vascular network. In the first days of plaque accumulation, a new immune cell profile is being formed, in which lymphocytes and macrophages join the neutrophils. This stage is followed by activation of the capillary bed resulting in the expansion of bacterial plaque, perivascular inflammatory infiltration, and vascular changes from early lesion. The production of gingival fluid increases and edema occurs in the gingival tissue [68]. As a result, normal angiogenic regulation is replaced by vasculature remodeling with vascular endothelial growth factor (VEGF) expressed from the periodontal pocket epithelium [69]. 

Vascular endothelial growth factor (VEGF) is a 45 kDa heparin-binding homodimeric glycoprotein with strong proangiogenic activity, and VEGF-A, VEGF-B, VEGF-C, VEGF-D, VEGF-E (virally encoded), and placental growth factor (PlGF) proteins are members of this family [64]. VEGF-A plays a dominant role in the regulation of angiogenesis. The binding of VEGF to endothelial cell-expressed receptor tyrosine kinases VEGFR-1 and VEGFR-2 stimulates endothelial cell migration, proliferation, and angiogenesis [65]. VEGF is expressed predominantly on vascular endothelial cells but can also be found on non-endothelial cells such as macrophages, keratinocytes, retinal pigmentary epithelial cells, bronchial epithelial cells, fibroblasts, tumor cells, and mast cells [70]. VEGF-A plays a dominant role in the regulation of angiogenesis. The binding of VEGF to endothelial cell-expressed receptor tyrosine kinases VEGFR-1, VEGFR-2, and a soluble form carrying only the ligand-binding region (sFlt-1/soluble VEGFR1) stimulate endothelial cell migration, proliferation, and angiogenesis [71]. VEGF gene expression is regulated through receptor-ligand balance and various signaling pathways such as phospholipase C-γ, protein kinase C, Ca2+, extracellular-signal-regulated protein kinase (ERK), Akt, Src, focal adhesion kinase, and calcineurin are involved in mediating multiple VEGF functions [72,73]. There is a cross-talk between these signaling pathways in pathological conditions such as inflammation, hypoxia, hypoglycemia, and cancer.

The main regulator of VEGF expression is the heterodimeric transcription factor hypoxia-inducing factor-1 (HIF-1), which binds to the VEGF gene-specific enhancer/promoter region. 

The decrease in oxygen tension suitable for cell physiology is the major trigger of oxygen depletion, angiogenesis, and VEGF activation against cellular stress. VEGF expression is induced by activation of HIF-1 in hypoxic tissues. Concomitantly, cellular stress causes inflammation to be triggered by the release of inflammatory cytokines and chemokines. It induces VEGF expression by the NF-κB pathway of inflammatory mediators such as IL-1, TNF-α, TGF-β. NF-κB and HIF-1 mutually up-regulate VEGF expression of inflammatory cytokines and mediators [74,75,76]. Hypoxic microenvironment and inflammation trigger the formation of ROS, causing activation of the COX-2-prostaglandin synthesis pathway [77]. This also activates the IL-1-NFκB-cyclooxygenase-2 (COX-2) axis bidirectionally and can up-regulate HIF-1α and induces VEGF [78]. 

Epidermal growth factor (EGF), platelet-derived growth factor (PDGF), epidermal growth, insulin-like growth factor, fibroblast growth factor, keratinocyte growth factor, up-regulate VEGF mRNA expression and are other factors responsible for VEGF regulation [71,78]. 

Numerous oncogenic mutations (vhl, ras, wnt-kras signaling pathway genes) are also involved with the regulation of VEGF expression. The AP-1 potential binding site sequences of c-fos family transcription factors involved in the transcription of proto-oncogenes are shown in the promoter region of the VEGF gene [71]. Therefore, oncogenic signaling pathways may be an expression inducer in VEGF regulation.

VEGF protein affects the vascular network during the progression of periodontal disease. Studies showed that tortuosity of vasculature and dilation increase with inflammation. Oral inflammation affects periodontal pocket formation through capillaries and venules in the early stages of the disease. There is a close relationship between vascular basement membrane thickening and increased blood vessels in the later stage of periodontal lesion formation [79]. Periodontitis is characterized by angiogenic changes within the periodontal tissues. These changes exacerbate periodontal inflammation and tissue damage by increasing the access of inflammation mediators and immune system cells to periodontal tissues.

**Figure 2:**
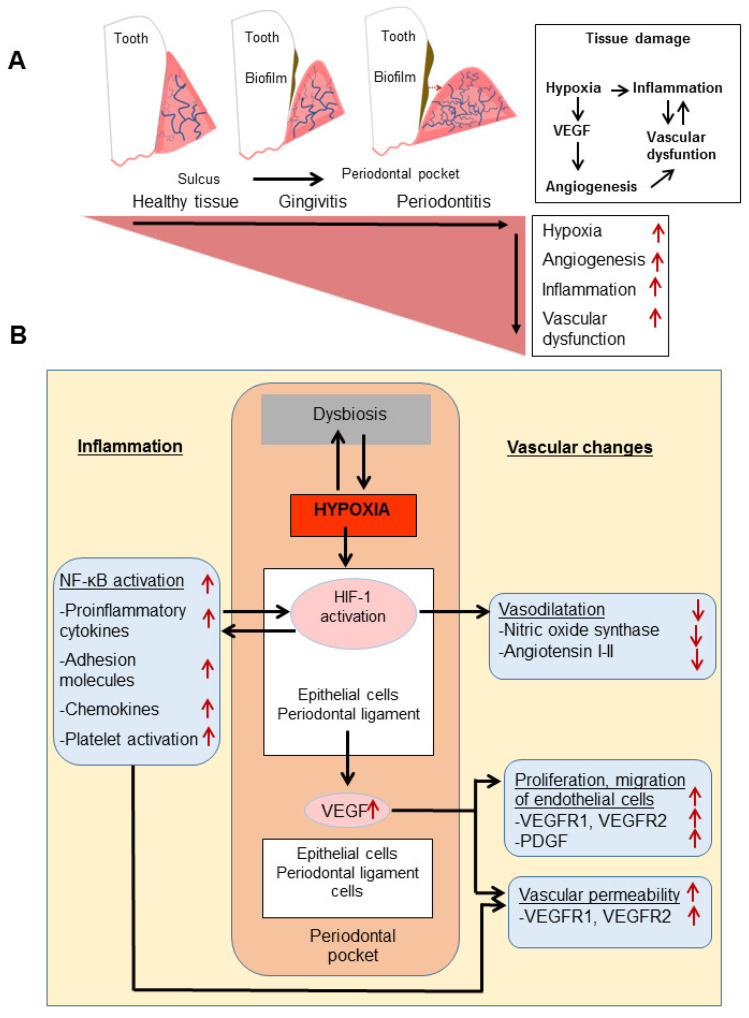
Effects of hypoxia and inflammation on the vascular network. Panel (**A**) shows hypoxia and inflammation-related angiogenesis in periodontal diseases. Controlling biofilm formation in healthy tissues and effective immune modulation prevents tissue from being exposed to destructive inflammation. In gingivitis, biofilm formation on the tooth surface creates a hypoxic microenvironment in the tissues and increases inflammation and angiogenesis. As a result, increased inflammation and angiogenesis lead to the formation of dental pockets, increased blood flow, edema, and vascular dysfunction. In the periodontitis stage, disrupted angiogenesis, enlarged periodontal pocket, and an expanded lesion are observed. As the angiogenesis becomes pathological, its effect causes vascular hemostasis dysfunction and uncontrolled inflammation. Panel (**B**) shows the effect of the hypoxic microenvironment in the periodontal pocket on the vascular network. The oxygen tension in the periodontal pocket is constantly changing. Oral pathogens exist in a gradient with time and changing hypoxic environment and undergo a dynamic colonization process. Hypoxia activates its primary mediator, the hypoxia-inducible factor (HIF-1), which is a transcription factor. VEGF expression from the epithelium and periodontal ligaments surrounding the vascular network in the periodontal pocket. At the same time, hypoxia induces inflammation via the NF-kB pathway. Both pathways lead to increased vascular permeability, increased vascularization, and decreased vascular bioavailability.

*P*. *gingivalis* can directly modulate endothelial cells and vascular networks by readily binding to endothelial cells and invading with its fimbriae. *P. gingivalis* also increases gene expression of various chemokines (e.g., CXCL8, CCL2), adhesion molecules [CD54, CD62E, PECAM-1 (Platelet endothelial cell adhesion molecule)], and inflammatory factors from endothelial cells [80,81,82] (Figure 2, Panel B). Gingipains, which are the proteolytic protein produced and release by *P. gingivalis*, modulate inflammatory mediators (ICAM-1/CD54, VCAM-1/CD106) expressed from endothelial cells and activate the kallikrein/kinin pathway [83,84]. *P. gingivalis*-derived gingipains mediate vascular damage by degrading endothelial PECAM-1 and VE-cadherin in vivo [9]. 

Increasing vascularity with angiogenesis in healthy tissues provides an advantage to the host for pathogen elimination. However, in chronic periodontitis, abnormal vascularization advances the severity of periodontal inflammation, as it facilitates the transport of more inflammatory cells, chemical mediators, and cytokines (Figure 1). Thus, disrupted angiogenesis is linked to tissue damage and uncontrolled inflammation in periodontitis [85]. Thus, the systemic effect of a disrupted and dysregulated angiogenesis, such as in rheumatoid arthritis, tumors, and ischemic vascular diseases, can be associated with uncontrolled angiogenic factors in the local periodontal tissue.

## 4. Hypoxia in Local Inflammation

Oxygen is essential for normal tissue functions, tissue development, and homeostasis. For the tissue to be defined as hypoxic, the amount of O_2_ required under normal conditions must be reduced. A medium containing 3–5% O_2_ is defined as moderate hypoxia, while cells give reversible and adaptive physiological responses to moderate hypoxia. In environments where oxygen tension drops to 0–1% O_2_ (low oxygen tension), cells may become dysfunctional and even apoptotic [86]. Except for embryogenesis, cell proliferation is low in healthy vascular tissues. Hypoxia enhances vascular development by stimulating multiple endothelial and stromal cells [87].

Hypoxia-inducible factor-1 (HIF-1) is a transcriptional mediator that is the primary regulator of O_2_ hemostasis and response to hypoxia. HIF-1 consists of a heterodimer of two subunits, HIF-1α and HIF-1β. HIF-1 is rapidly degraded under normoxic conditions via ubiquitination and subsequent proteasomal degradation. HIF-1a subunit has two conserved proline residues (P402 and P564), which are oxygen- and iron-dependent prolyl hydroxylases (PHDs) under normoxia [88]. Hypoxia inactivates PHDs, stabilizing the HIF-1α subunit, and HIF1α translocates from the cytosol to the nucleus. The inactivation of PHDs activates HIF-1 in the presence of iron and oxygen deprivation. It is inactive in the cell under normoxic conditions. Under hypoxic conditions, it is activated and causes the expression of proangiogenic factors such as nitric oxide synthase (NOS), VEGF, pro-inflammatory cytokines, erythropoietin, endothelin 1, placental growth factor (PIGF), and angiopoietins [89,90,91,92]. 

HIF-1 and NFκB are the most effective signaling pathways in the response of cells and tissues to stress, which have major roles in hypoxia and inflammation. Cross-talk connections between these two pathways lead to synergistic relationships involving co-activating stimuli and shared regulators and targets. The main activator-regulatory protein for NF-κB, IKK–TGFβ, is identified as a common general activator protein and TAK-dependent manner induce in both NF-κB and HIF-1 pathways in hypoxia. It has also been shown that NF-κB directly modulates HIF-1 expression by binding to the HIF-1 gene region in inflammation and hypoxia [93]. In addition, many signaling pathways play a role in HIF-1 regulation in inflammation. In the decreasing oxygen gradient due to inflammation, immune cells trigger HIF-1 activation in the area of infection characterized by hypoxia. After LPS stimulation, p44/42 mitogen protein kinases (MAPK) pathway for macrophage-monocyte Cell is activated, increasing HIF-1 binding to HRE sequences [94] and LPS-TLR4 binding induce HIF-1 activation in human gingival fibroblasts [95]. On the other hand, TLR signaling induces pro-inflammatory cytokine production by NF-κB activation from the cell. In addition to stimulating the inflammatory response, NF-κB also induces a response to hypoxia by modulation of HIF-1. For example, *P. gingivalis* and its LPS activates the signaling of NF-κB and HIF-1 by hypoxia of periodontal ligament cells and stimulates the expression of VEGF, IL-1 [96] (Figure 2), and ROS formation [97]. *F. nucleatum* increases the secretion of pro-inflammatory cytokines (TNF-α, IL-1) in hypoxic gingival tissues and induces NF-kB-dependent NOS synthesis [98]. Although HIF-1 activation via LPS has been demonstrated experimentally, the relationship between NF-kB (pro-inflammatory cytokine synthesis pathway)-LPS-HIF-1 interaction mechanism still needs to be determined.

Degranulation of neutrophils in inflammation causes the accumulation of reactive oxygen radicals in the microenvironment. In addition, thrombin and blood coagulation factors, which coordinate changes in the vascular wall caused by inflammation, stimulate the production of reactive oxygen radicals. It has been shown that ROS products trigger HIF-1 activation and VEGF production in human vascular smooth muscle cells [99]. Another regulation pathway of HIF-1 is its induction through the PI3K/mTOR signaling pathway during T cell stimulation. During inflammation, T cell receptor TCR-PI3K/mTOR signaling in a hypoxic environment causes HIF-1 stabilization and activation of HIF-1 protein [100]. HIF-1 can also be induced indirectly, non-hypoxically, by activating angiotensin I and II, which regulate vascular tone. Angiotensin I and angiotensin II are mediators produced by tissue cells through intracellular RAS expression and by mitogen-activated protein kinases (MAPK) and phosphoinositide-3 kinase (PI3K) pathways. The angiotensin-binding receptors of AT1R and AT2R are found in both arteries and veins and are involved in the regulation of vascular tone. The binding of angiotensin II to AT1R produces a pro-inflammatory effect on endothelial cells and vascular smooth muscle cells. It induces the synthesis of adhesion molecules, cytokines, and chemokines of vascular cells. AT1R activation also increases NO synthesis and vascular relaxation. AT2R induces the release of vasoactive peptides. It also causes inflammation by increasing fibrosis, oxidative stress, and ROS production. There are only a few studies on the effect of this pathway in periodontal diseases. RAS components are detected in periodontal tissues in both humans and mice. In periodontal diseases, endothelial cells and other gingival cell types such as epithelial cells produce large amounts of renin [101]. ANRI and ANRII expressions and angiotensin-converting enzyme (ACE) activity are increased in the tissues of periodontitis patients [102]. These limited findings show the effect of RAS activation on the local vascular network in periodontal diseases. Their relevance in the relationship with the systemic vascular network and systemic diseases needs to be clarified.

It has been shown that angiopoietin-like 4, an important signaling factor in modulation of angiogenesis, inflammation, lipid metabolism, and bone resorption, is induced by HIF-1 in human periodontal ligament fibroblasts and it can also be modulated by *P. gingivalis* LPS, IL-1 and TNF-α [103]. Although the function of angiopoietin-like-4 in periodontal tissues is not yet fully known, its mediation of angiogenesis and bone regeneration in inflammation, especially under hypoxic conditions, indicates that it may stand out as a good therapeutic agent candidate for periodontal diseases.

A synergistic effect of oxygen depletion and tissue inflammation on the vessel may be the most critical factor in the chronicity of periodontitis and its spread to the system. In both cases, the vascular network that manages and regulates inflammation is under heavy impact, where the hypoxic environment may be both a cause and a consequence of inflammation (Figure 2). 

During periodontal inflammation, the dental pocket is exposed to a complex microbiome of aerobic and anaerobic bacteria. Biogeography of the microbiome is central for forming and spreading predominant bacterial colonization in the oral cavity [104]. There is a temporal component of bacterial expansion and site-specific colonization in the niches of the oral cavity. In this model, different areas in the oral cavity, tooth shape, and location of dentition, oral microenvironmental factors, host genetic characteristics act as determinants where the colonization may be possible when a hierarchical group adapts to changing conditions becomes dominant. 

The oxygen tension gradient in the dental pocket suggests a dynamic bacterial colonization and a variable composition according to hypoxia change. As a result of the reduction of tissue oxygen tension, the colonies forming the biofilm evolve from aerobic bacterial groups to anaerobic bacterial groups. Periodontal bacteria such as *F. nucleatum* and *P. gingivalis* may directly manage hypoxic responses and colonization in the dental pocket. *F. nucleatum* causes endothelial cell dysfunction by inducing hypoxia and thus may contribute to periodontitis pathology [105,106]. IL-1-mediated caspase 1 activity of *P. gingivalis*, which is defined as the key pathogen of bacterial colonization, was investigated under normoxia and hypoxia conditions. The NLRP3 inflammasome increased the secretion of IL-1β, IL-6, and TNF-α in human periodontal ligament fibroblasts, and Gram-negative bacteria LPS have been shown to activate the mechanism. However, in intense and uncontrollable inflammation, IL-1-mediated caspase 1 activation may be responsible for cell death. In experimental periodontitis studies, the effects of hypoxia and LPS on NLP3 inflammasome activation were investigated. The synergistic effect of hypoxia and *P. gingivalis*-LPS induced IL-1 release stimulated an inflammatory response that would cause pyroptosis in human periodontal ligament fibroblasts [107]. *P. gingivalis* and *E. coli* LPS activated the inflammatory response and the caspase 1 cascade in gingival fibroblasts. Interestingly, at an early stage, unlike *P. gingivalis*, *E. coli* LPS transformed the normoxic phase into the hypoxia phase [108]. This is still an understudied area where spatiotemporal bacterial colonization associated with unstable oxygen tension in the dental pocket needs to be elucidated.

Tissue oxygen tension is regulated by angiogenesis, which increases tubular formation—the hypoxic microenvironment impacts angiogenesis, one of the critical mechanisms of homeostasis, in turn. The proangiogenic factor VEGF is central to this bidirectional link. Under hypoxic conditions, VEGF regulates neovascularization, expands the vascular network, and induces angiogenesis in destroyed periodontal tissues, which may not be an organized and homeostatic event and leads to granulation tissue formation. Endothelial cells increase VEGFR expression in a hypoxic environment induced by the *F. nucleatum* and induce TNF-α and NOS. Hypoxia is suppressed by CD31 and CD34, which are endothelial cell surface markers [105]. Both markers are known to be associated with cell-cell binding. Thus, hypoxia is critical for increased vascular permeability causing endothelial dysfunction by suppressing adhesion molecules [109] (Figure 2).

## 5. Conclusions

The homeostatic dynamics of the oral cavity are constantly changing with microenvironmental conditions, histological differentiation, and contact of host and oral microorganisms. Oral pathogen dissemination is countered with host immune cell function with a delicate balance between the host and microbes under homeostatic conditions. Therefore, periodontal health is not a sterile environment where the microbes and other microorganisms are entirely missing. An ongoing balanced activity determines the cooperation and escape strategies of pathogens and the response of immune system cells. The immune system is also responsible for repairing damaged tissue along with microbial elimination. Although local inflammation does not have a devastating effect initially, it can become chronic within exacerbated microbial invasion and trauma and cause secondary tissue damage in the periodontium. Tissue damage and vascular changes come to the fore as determining factors at the center of the host-pathogen relationship.

On the one hand, constant bacterial invasion, and on the other hand, the destructive effect of inflammation causes unstable oxygen tension and reactive oxygen radical accumulation in the periodontium, especially in the dental pocket. The prolonged hypoxia can lead to vascular dysfunction, especially in endothelial cells. Hypoxia can affect the expression profile of surface markers (CD31, CD34) responsible for vascular tubule formation, cell-cell interaction, and differentiation [105]. In addition, dysfunctional endothelial cells can exacerbate local inflammation. Decreased expression of CD31 and increased expression of CD34 may impair vascular integrity and may allow pro-inflammatory cytokines and oral antigens to enter the circulation.

Another effect of prolonged hypoxia in tissues is the promotion of critical regulators of neovascularization VEGF, VEGFR1, and VEGFR2. Therefore, pathological vascularization may be the initiating step of the inevitable inflammatory cascade in the pathological process of periodontal disease.

The relationship between periodontal diseases and systemic inflammatory diseases includes uncontrolled inflammation. The interaction of distant tissues and organs via the vascular network and common inflammatory mediators, which may trigger the systemic inflammation in the circulation, make this link even more probable. Studies have shown a link between cardiovascular diseases, diabetes, rheumatoid arthritis, Alzheimer’s disease, and periodontal disease pathologies. Although organ and tissue-specific cellular and molecular mechanisms need to be clarified, similar vascular changes may occur in the pathologies of systemic inflammation-related diseases. Hence, we need to better understand the vascular effects of periodontal diseases at the local and systemic levels to establish novel hypotheses, find strategic methods to control periodontal disease-associated systemic diseases, and develop therapeutic approaches.

## Data Availability

The data that supports the findings of this study are available within the article.
